# Supplementary material for: Visualizing trypanosomes in a vertebrate host reveals novel swimming behaviours, adaptations and attachment mechanisms
Source: eLife. 2019 Sep 24;8:e48388. doi: 10.7554/eLife.48388 (PMC6759355; doi:10.7554/eLife.48388)
Supplement: Transparent reporting form [file elife-48388-transrepform.pdf]

1st Floor
24 Hills Road
Cambridge CB2 1JP, UK

P 01223 855340
W elifesciences.org
T @elife

eLife's transparent reporting form

We encourage authors to provide detailed information *within their submission* to facilitate the interpretation and replication of experiments. Authors can upload supporting documentation to indicate the use of appropriate reporting guidelines for health-related research (see EQUATOR Network), life science research (see the BioSharing Information Resource), or the ARRIVE guidelines for reporting work involving animal research. Where applicable, authors should refer to any relevant reporting standards documents in this form.

If you have any questions, please consult our Journal Policies and/or contact us: editorial@elifesciences.org.

Sample-size estimation

- You should state whether an appropriate sample size was computed when the study was being designed
- You should state the statistical method of sample size computation and any required assumptions
- If no explicit power analysis was used, you should describe how you decided what sample (replicate) size (number) to use

Please outline where this information can be found within the submission (e.g., sections or figure legends), or explain why this information doesn't apply to your submission:

Our study mostly relies on high-speed videos capturing the swimming behavior of trypanosomes *in vivo*. This is the first report of such kind, so we could not predict the number and type of observations we would report. Nevertheless, every time we observed something, as for example trypanosome attachment through the posterior end or an alternative way of inverting direction, we captured and reported the phenomenon in several locations within the host and at different time points after infection. This assured that we could make a general statement about the swimming behavior of this trypanosome in this vertebrate host.

Furthermore, the advantage of the *in vivo* imaging is that we could rely on the behavior of several trypanosomes being captured within a recording period (usually between 10-20 second) and had to make no assumptions about culture media, temperature, or environment, since it was recorded in a natural host of the trypanosome.

As for the data in figure 1, reporting on the type of swimming behavior and the speed of swimming, we again had no previous reference for this trypanosome. In our daily observations however, we consistently observed that the majority of the trypanosomes were tumblers, but that occasionally, intermediate, and more seldomly, persistent swimmers, could be observed. For this reason, to estimate for the first time the proportion of tumblers, intermediate or persistent swimmers, we captured and analyzed the swimming behavior of almost 1000 *T. carassii*. these details are also reported in the manuscript.

Replicates

- You should report how often each experiment was performed
- You should include a definition of biological versus technical replication
- The data obtained should be provided and sufficient information should be provided to indicate the number of independent biological and/or technical replicates
- If you encountered any outliers, you should describe how these were handled
- Criteria for exclusion/inclusion of data should be clearly stated
- High-throughput sequence data should be uploaded before submission, with a private link for reviewers provided (these are available from both GEO and ArrayExpress)

Please outline where this information can be found within the submission (e.g., sections or figure legends), or explain why this information doesn't apply to your submission:

For *in vitro* imaging and analysis of trypanosome swimming behavior (figure 1A), we considered a biological replicate the isolation of *T. carassii* from different carp infections; For the data reported in figure 1, we imaged and recorded the swimming behavior of a total number of 944 *T. carassii*, isolated from 6 different carp infections and imaged over 60 independent acquisitions.
For the quantification of trypanosome speed and length (figure 1B-C), each trypanosome was considered a biological replicate.
For the analysis of trypanosome length, 10 freshly isolated trypanosomes obtained from 4 independent infections were analyzed. For each of them, more than 20 frames within the same acquisition (technical replicates) were used to calculate the average length of one trypanosome.
For the analysis of speed, each trypanosome was considered a biological replicate; tracks of n=16 tumblers, n=14 intermediates, and 1 persistent, randomly selected from the 944 trypanosomes analyzed in figure 1A, were generated using ImageJ. For each trypanosome, the program provides the average speed between two consecutive points on a track, and each track was composed of a minimum 25 to a maximum of 82 points, depending on the recording time (10-20 seconds) and acquired frames per second (100-240 fps). The speed at each point was considered a technical replicate within each biological sample.

For the *in vivo* analysis, each individual being imaged (either infected or non-infected), was considered a biological replicate. Our *in vivo* imaging relied on the acquisition and analysis of approximately 224 high-speed videos.

For the analysis in figure 8, the caudal vein diameter of n=10 non-infected and n=16 infected individuals was analyzed (biological replicated). Each value is the average of at least 3 measurements (technical replicates) taken at different locations within the caudal vein of the same individual.

Statistical reporting

- Statistical analysis methods should be described and justified
- Raw data should be presented in figures whenever informative to do so (typically when N per group is less than 10)
- For each experiment, you should identify the statistical tests used, exact values of N, definitions of center, methods of multiple test correction, and dispersion and precision measures (e.g., mean, median, SD, SEM, confidence intervals; and, for the major substantive results, a measure of effect size (e.g., Pearson's r, Cohen's d)
- Report exact p-values wherever possible alongside the summary statistics and 95% confidence intervals. These should be reported for all key questions and not only when the p-value is less than 0.05.

Please outline where this information can be found within the submission (e.g., sections or figure legends), or explain why this information doesn't apply to your submission:

N/A

(For large datasets, or papers with a very large number of statistical tests, you may upload a single table file with tests, Ns, etc., with reference to sections in the manuscript.)

Group allocation

- Indicate how samples were allocated into experimental groups (in the case of clinical studies, please specify allocation to treatment method); if randomization was used, please also state if restricted randomization was applied
- Indicate if masking was used during group allocation, data collection and/or data analysis

Please outline where this information can be found within the submission (e.g., sections or figure legends), or explain why this information doesn't apply to your submission:

N/A

Additional data files ("source data")

- We encourage you to upload relevant additional data files, such as numerical data that are represented as a graph in a figure, or as a summary table
- Where provided, these should be in the most useful format, and they can be uploaded as "Source data" files linked to a main figure or table
- Include model definition files including the full list of parameters used
- Include code used for data analysis (e.g., R, MatLab)
- Avoid stating that data files are "available upon request"

Please indicate the figures or tables for which source data files have been provided:

Figure 1A-B
Figure 2
Figure 8

eLife Sciences Publications, Ltd is a limited liability non-profit non-stock corporation incorporated in the State of Delaware, USA, with company number 5030732, and is registered in the UK with company number FC030576 and branch number BR015634 at the address 1st Floor, 24 Hills Road, Cambridge CB2 1JP | August 2014
